# Isoprenoid Metabolism and Engineering in Glandular Trichomes of *Lamiaceae*

**DOI:** 10.3389/fpls.2021.699157

**Published:** 2021-07-19

**Authors:** Soheil S. Mahmoud, Savanna Maddock, Ayelign M. Adal

**Affiliations:** Department of Biology, The University of British Columbia, Kelowna, BC, Canada

**Keywords:** glandular trichomes, metabolic engineering, isoprenoids, mint, lavender

## Abstract

The isoprenoids play important ecological and physiological roles in plants. They also have a tremendous impact on human lives as food additives, medicines, and industrial raw materials, among others. Though some isoprenoids are highly abundant in nature, plants produce many at extremely low levels. Glandular trichomes (GT), which cover the aerial parts of more than 25% of vascular plants, have been considered as natural biofactories for the mass production of rare industrially important isoprenoids. In several plant genera (e.g., *Lavandula* and *Mentha*), GTs produce and store large quantities of the low molecular weight isoprenoids, in particular mono- and sesquiterpenes, as essential oil constituents. Within each trichome, a group of secretory cells is specialized to strongly and specifically express isoprenoid biosynthetic genes, and to synthesize and deposit copious amounts of terpenoids into the trichome’s storage reservoir. Despite the abundance of certain metabolites in essential oils and defensive resins, plants, particularly those lacking glandular trichomes, accumulate small quantities of many of the biologically active and industrially important isoprenoids. Therefore, there is a pressing need for technologies to enable the mass production of such metabolites, and to help meet the ever-increasing demand for plant-based bioproducts, including medicines and renewable materials. Considerable contemporary research has focused on engineering isoprenoid metabolism in GTs, with the goal of utilizing them as natural biofactories for the production of valuable phytochemicals. In this review, we summarize recent advances related to the engineering of isoprenoid biosynthetic pathways in glandular trichomes.

## Isoprenoid Diversity and Biosynthesis

The isoprenoids or terpenoids, make up the largest class of plant secondary, or specialized metabolites. They play crucial ecological roles as pollinator attractants and defensive agents, and have important physiological functions as plant hormones and photosynthetic pigments, among others (Gershenzon and Dudareva, 2007; Okada, 2011; Tetali, 2018). Isoprenoids impact human lives through imparting scent, flavor, and health-promoting properties to fruits, vegetables, and medicinal plants (Cordell and Colvard, 2012). Some terpenoids have potent biological activities, and have applications as prescription drugs and over-the-counter medicines. A few have also been used as sustainable replacements for petroleum-derived chemicals (Cordell and Colvard, 2012; Tetali, 2018).

**Figure 1:**
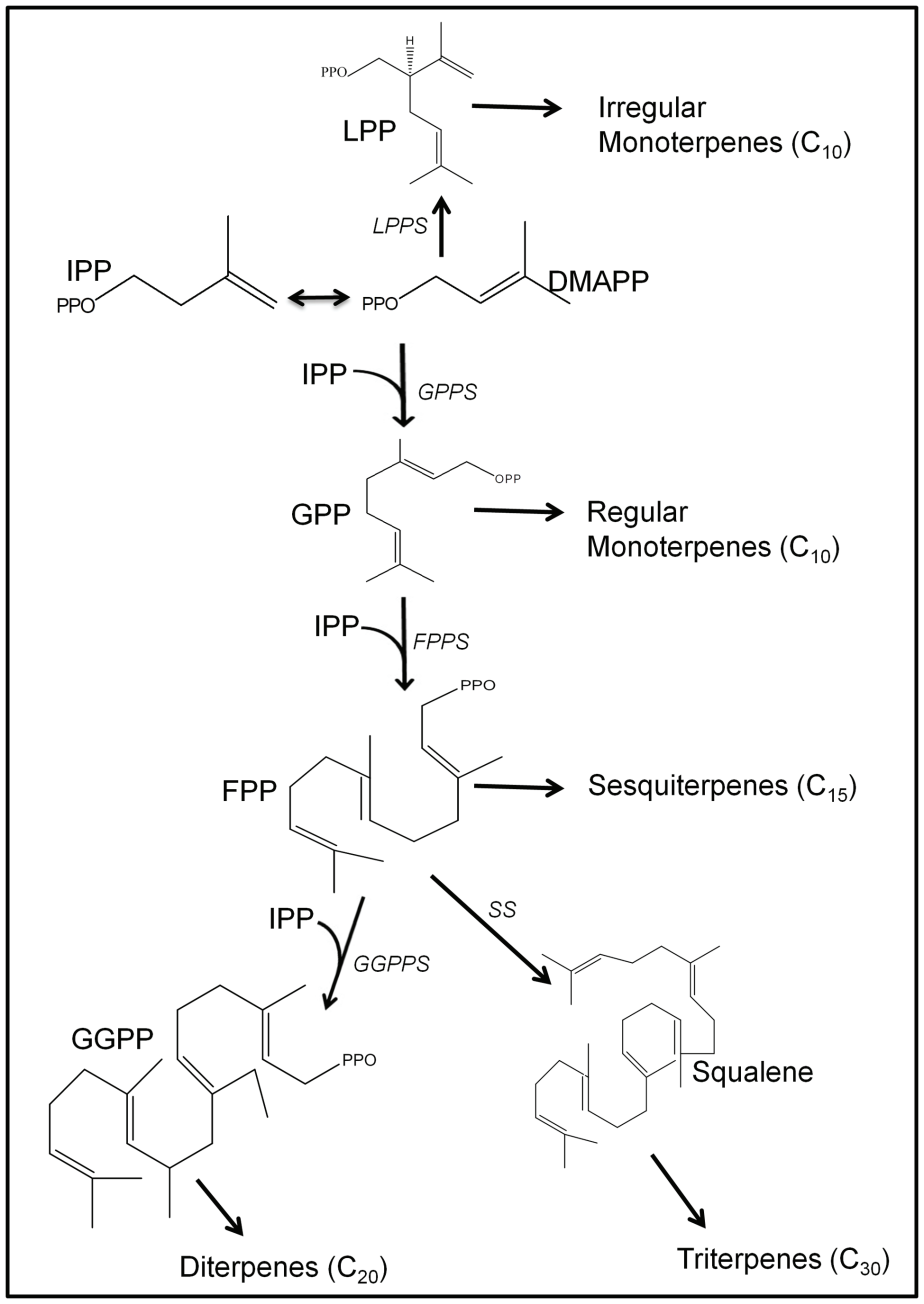
Overview of the biosynthetic pathways for major plant isoprenoids. The biosynthesis of isoprenoids starts with a common pool of isopentenyl diphosphate (IPP) and dimethylallyl diphosphate (DMAPP). IPP and DAMPP are initially condensed to form either lavandulyl diphosphate (LPP), the precursor for irregular monoterpenes, or geranyl diphosphate (GPP), the precursor to regular monoterpenes. GPP can also be condensed with one or two IPP units to form FPP for sesquiterpene synthesis, or geranylgeranyl diphosphate (GGPP) for diterpene production. Two FPP units can be condensed to form squalene, the immediate precursor for triterpene metabolism. The isoprenyl diphosphate synthases (IDSs) that catalyzed the precursor(s) into specific prenyl diphosphate include LPP synthase (LPPS), GPP synthase (GPPS), geranylgeranyl diphosphate synthase (GGPPS), FPP synthase (FPPS), and squalene synthase (SS).

Isoprenoids are synthesized through the condensation of isopentenyl diphosphate (IPP; C_5_) and its isomer dimethylallyl diphosphate (DMAPP; C_5_), and are classified by the number of five-carbon units present in the core structure (Bouvier et al., 2005; Defilippi et al., 2009). Major isoprenoid classes include monoterpenes (C_10_), sesquiterpenes (C_15_), diterpenes (C_20_), triterpenes (C_30_), and tetraterpenes (C_40_), although lower and higher-order isoprenoids (e.g., isoprene and natural rubber, respectively) also exist (Figure 1). In general, the biosynthesis of isoprenoids can be classified into the following four states: generation of general precursors IPP and DMAPP, production of specific isoprenyl diphosphates for various isoprenoid classes, the transformation of isoprenyl diphosphates to individual isoprenoids by terpene synthase (TPS) enzymes, and structural modifications catalyzed by other catalysts (Figure 1).

In plants, IPP and DMAPP are generated through two distinct compartmentally separated pathways. In plastids, the 1-DXP pathway, aka MEP pathway, produces IPP/DMAPP for monoterpene, diterpene, and tetraterpene biosynthesis (Lois et al., 2000; Turner et al., 2000). In the cytosol, the classical mevalonic acid (MVA) pathway supplies IPP, which is isomerized to DMAPP by an isomerase. The cytosolic IPP/DMAPP pool is primarily used for the production of sesqui- and triterpenes (Laule et al., 2003). All biochemical steps of both pathways have been characterized, and the relevant genes cloned (Tholl and Lee, 2011).

The head-to-tail condensation of IPP and DMAPP yields various linear isoprenoid precursors, including geranyl diphosphate (GPP; C_10_), neryl diphosphate (NPP; C_10_), farnesyl diphosphate (FPP; C_15_), and geranylgeranyl diphosphate (GGPP; C_20_). The head-to-middle condensation of two DMAPP units also occurs in some plants, and gives rise to lavandulyl diphosphate (LPP; C_10_) as a linear product. The condensation reactions are catalyzed by a group of enzymes known as isoprenyl diphosphate synthases (IDSs; Ogura and Koyama, 1998; Burke et al., 1999; Schilmiller et al., 2009; Tholl and Lee, 2011; Demissie et al., 2013; Dudareva et al., 2013; Adal and Mahmoud, 2020). IDSs are classificed into “*cis*” or “*trans*” based on their primary structures, and the stereochemistry of the products they generate (Nagel et al., 2019). The *trans*-IDSs catalyze the synthesis of the common terpene precursors, and are distinguished by two conserved aspartate-rich motifs, DDX_2–4_D and (N/D)DX_2_D, which serve as substrate and divalent metal ion cofactor binding sites. On the other hand, *cis*-IDSs lack these conserved motifs, and instead share five conserved regions designated as Regions I–V, including catalytically essential aspartate residue in Region IV and the glutamate residue in Region V (Kharel and Koyama, 2003). For example, GPPS, a typical *trans*-IDS, catalyze the head-to-tail condensation of DMAPP with IPP to produce GPP, while LPPS, a *cis*-IDS, generates LPP through the head-to-middle condensation of two DMAPP units in lavenders (Demissie et al., 2013; Adal and Mahmoud, 2020).

Other *trans*-IDSs include FPP, which gives rise to sesquiterpenes and triterpenes, and GGPP, which serves as a precursor for the synthesis of diterpenes and tetraterpenes. The prenyl diphosphates are transformed into various terpenoids by specific TPSs, of which hundreds have been cloned from a wide range of plants.

## Glandular Trichomes

**Figure 2:**
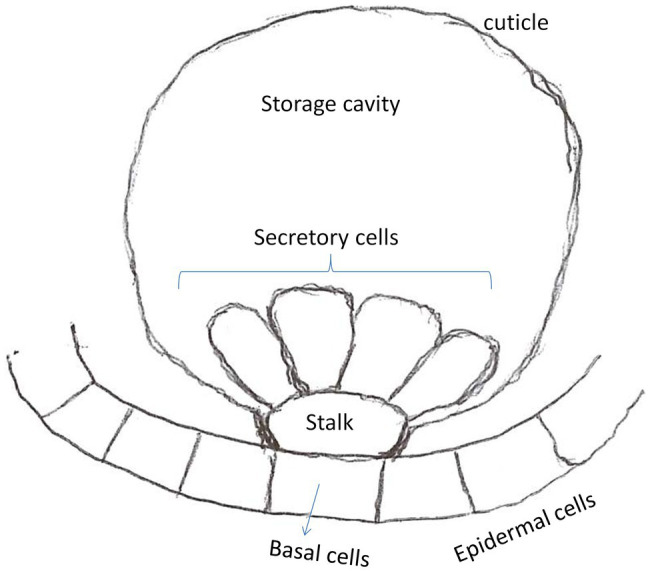
Schematic representation of peltate glandular trichome found in *Lamiaceae* plants. The secretory cells produce and secret essential oil constituents into the trichome’s secretory cavity.

Glandular trichomes (GTs) can be found on the surfaces of leaves, stems, petals, sepals, petioles, peduncles, and seeds of ca. 30% of vascular plants (Fahn, 2000; Wagner et al., 2004; Glas et al., 2012). Regardless of their location on the plant, GTs are multicellular structures, each consisting of a basal cell, one or more stalk cells, and a group of 4–8 (depending on species) secretory cells (Figure 2; Fahn, 2000; Werker, 2000; Schnittger and Hülskamp, 2002; Huchelmann et al., 2017). A typical secretory cell is non-photosynthetic, and is specialized to produce relatively large amounts of specialized metabolites (Fahn, 2000). In general, two types of glandular trichomes – capitate GT and peltate GT – can often be found in several plant families, including *Lamiaceae* and *Solanaceae*. Both GT types share certain structural features (Fahn, 2000). For example, both include a basal cell, a stalk that can be made up of one to several cells, and a group of secretory cells that are clustered together on the apex of the stalk. However, they produce different classes of metabolites. The capitate GTs mainly produce non-volatile compounds, that are not stored in the trichome but are mostly exuded and accumulate on the surface of the trichome (Glas et al., 2012). Peltate GTs, on the other hand, develop a storage cavity capable of storing relatively large quantities of primarily volatile compounds (Glas et al., 2012; Huchelmann et al., 2017). The latter are responsible for the production and storage of essential oil constituents in members of the *Lamiaceae*, including peppermint, spearmint, lavender, basil, and so forth.

## Engineering Isoprenoid Biosynthesis in Trichomes

Several platforms, including bacteria (Farrokh et al., 2019), yeast (Rahmat and Kang, 2020), algae (Rico et al., 2017), and plants (Birchfield and McIntosh, 2020), have been successfully used for enhanced (or mass) production of specific isoprenoids of industrial value. Though traditionally microbial systems have been the platform of choice for the production of recombinant natural products, plants are emerging as important alternatives. This is in part due to the relative ease and cost-effectiveness of mass-producing plants in general, and in part due to the fact that genetic transformation of most plants is now routine. Plants bearing GTs are particularly suited for mass production of secondary metabolites, as GTs have specialized secretory cells, capable of production and secretion of large quantities of phytochemicals (that are often toxic to other cell types) into the GT’s storage cavity. In this context, several GT-bearing plants have been investigated as potential biofactories for the mass production of specific metabolites, with most of the studies reported focussing on the plants discussed below.

### Mints (*Mentha*)

**Figure 3:**
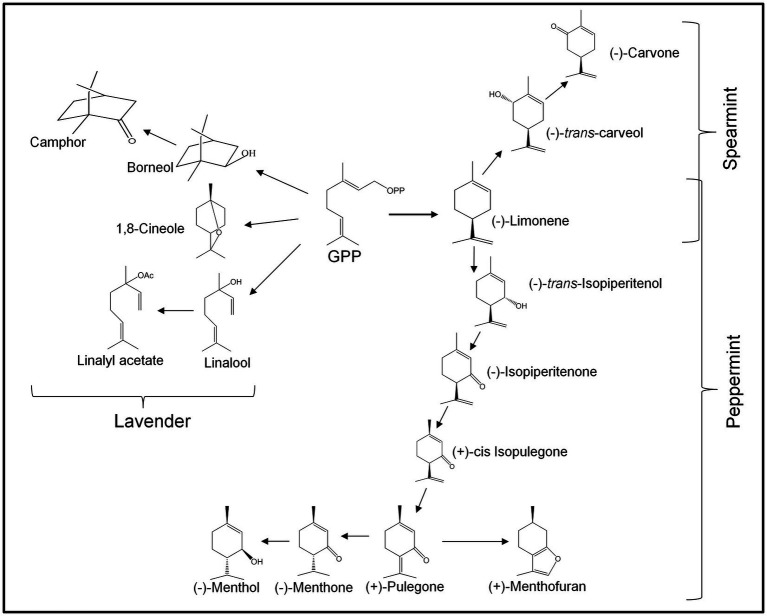
The biosynthetic pathways for monoterpene metabolism in lavender, peppermint, and spearmint. Representative monoterpenes derived from GPP are shown, and the corresponding enzymes involved in the process are previously reported for each species (Croteau et al., 2005; Ringer et al., 2005; Landmann et al., 2007; Demissie et al., 2012; Adal et al., 2019).

Metabolic engineering to enhance isoprenoid (essential oil monoterpenes) metabolism has been reported in the two most commercially important mint species, *Mentha x piperita* (peppermint) and *Mentha spicata* (spearmint), which are widely grown for EO production. The EO in these plants is a complex mixture of mainly monoterpenes, many of which are highly abundant while others are present in trace quantities. In both species, GPP is initially converted to (−)-limonene through a reaction catalyzed by the enzyme (−)-limonene synthase (Figure 3). In peppermint, hydroxylation at the C-3 position of the limonene ring initiates a cascade of reactions that lead to the production of several monoterpenes, of which (−)-menthol is highly valued for its applications in food and alternative medicine. In spearmint, hydroxylation occurs on the C-6 position of limonene, leading to the production of (−)-*trans*-carveol, which is efficiently oxidized to (−)-carvone as the main EO constituent.

One of the earliest metabolic engineering efforts in mint was reported in 2001, in a study that attempted to increase flux toward monoterpene metabolism by overexpressing the coding sequence for the branch-point enzyme 4S-limonene synthase (*4S-LimS*) in transformed *M. x piperita* plants (Diemer et al., 2001). The expression of *4S-LimS* transgene was driven by the Cauliflower Mosaic Virus (CaMV) 35S promoter. Although monoterpene production was not altered in most plants, a few transformants accumulated higher levels of essential oil constituents, including 56% more pinene, 22–74% higher 1,8-cineole, and 18–40% more pulegone than control plants. A few attempts followed this investigation in Professor Rodney Croteau’s laboratory in the early 2000s. In one attempt, the DXP reductoisomerase (DXR) cDNA (Figure 1) was overexpressed in peppermint plants to enhance the output of the DXP pathway, and improve the production of precursor (IPP/DMAPP) for monoterpene biosynthesis (Mahmoud and Croteau, 2001). Several transgenic plants produced up to 50% more EO than the wild-type controls. In the same study, the menthofuran synthase gene, responsible for the production of the undesired oil monoterpene constituent (+)-Menthofuran (Figure 3), was overexpressed in separate peppermint plants in sense and antisense to evaluate the effects of these overexpressions on menthofuran production (Mahmoud and Croteau, 2001). Most plants expressing the transgene in sense accumulated substantially more menthofuran than wild-type controls. Conversely, plants that overexpressed the gene in antisense orientation, accumulated significantly less menthofuran than control plants. Intriguingly, menthofuran production was almost entirely eliminated in plants in which the menthofuran synthase gene was co-suppressed. Surprisingly, growth and development in co-suppressed plants were compromised as some of the most affected plants appeared bleached, in particular, under stress conditions. Another study that aimed to enhance monoterpene production (oil yield) in peppermint, limonene synthase, and limonene-3-hydroxylase cDNAs were overexpressed in independently (separately) transformed peppermint plants (Mahmoud et al., 2004). Overexpression of either gene failed to improve oil yield significantly. However, limonene levels increased dramatically, surpassing 80% of total oil (compared to ~2% in wild-type plants), in the transformed peppermint lines, in which limonene-3-hydroxylase was co-suppressed. Taken together, studies in peppermint produced evidence that metabolic engineering may be employed to effectively engineer isoprenoid metabolism. However, not all metabolic steps respond to gene overexpression, while gene co-suppression can effectively eliminate unwanted metabolites (e.g., menthofuran) or enhance the accumulation of particular desired end products (e.g., limonene).

Modulating the expression of transcription factors that regulate other EO biosynthetic genes offers another avenue for enhancing isoprenoid production in GTs. A recent study investigated the effects of overexpressing and silencing the spearmint GT-specific transcription factor MsYABBY5 on the production of terpenes in spearmint (Wang et al., 2016). The TF was constitutively overexpressed in stably transformed plants, and silenced using RNA interference (RNAi). Silencing MsYABBY5 increased the levels of terpenes in spearmint from 20 to 77%, while its overexpression led to a 23–52% decrease in the overall levels of terpenes in spearmint plants. This study clearly demonstrated that the MsYABBY5 is a repressor of monoterpene metabolism in spearmint, and that manipulating the expression of transcription factors offers a viable method for enhancing terpene production in plant GTs. In a separate study, the expression of the spearmint GT-specific R2R3-MYB TF was suppressed by RNAi (Reddy et al., 2017). Likewise, suppression of MsR2R3-MYB expression led to a 2.3–4.5-fold increase in total monoterpene abundance in transgenic spearmint. It was further shown that MsR2R3-MYB suppresses the expression of the GPP synthase large subunit gene through binding of its cis-elements, and hence acts as a negative regulator of monoterpene metabolism in spearmint.

A recent study evaluated the potential of spearmint for the production of heterologous monoterpene through metabolic engineering. In this study, a transgenic spearmint line with reduced limonene and carvone was independently transformed with cDNAs of linalool synthase and myrcene synthase from *Picea abies*, and geraniol synthase from *Cananga odorata*, which were also controlled by 35S promoter. Silencing of the limonene synthase gene by RNAi led to a huge reduction in the production of limonene and carvone, and an increase in sesquiterpene, phytosterols, fatty acids, flavonoids, and phenolic metabolites. Surprisingly, overexpression of heterologous TPSs in these lines did not significantly increase the production of the related products, although small quantities of some heterologous terpenes were produced, which were oxygenated by the host plant (Li et al., 2020).

### Lavenders (*Lavandula*)

Lavenders (*Lavandula*) are perennial members of the mint family. To date, ca. 34 species have been described, three of which (*L. angustifolia*, *L. latifolia*, and their natural hybrid offspring *L. x intermedia*) are widely grown for EO production. Between 1,200 and 1,500 tons of lavender, EO is produced worldwide per annum for use in foods, cosmetics, and personal care products. As in other members of the Labiatae, lavender bears peltate GT (Huang et al., 2008) that produce and store relatively large quantities of a monoterpene-rich essential oil.

The EO of a typical lavender species consists of over 50 monoterpenes, the most abundant of which include linalool, linalool acetate, borneol, camphor, and 1,8-cineole. Among these, camphor, linalool, and linalool acetate are key determinants of the quality (scent and bioactivity) of lavender EO (Lis-Balchin, 2002; Upson and Andrews, 2004). Lavender EOs with high linalool and linalool acetate, and low borneol, camphor, and 1, 8-cineole content are considered to be of “high quality,” and are used in cosmetic products and aromatherapy (Cavanagh and Wilkinson, 2002). These oils are typically obtained from true lavender (*L. angustifolia*) species. Oil marketed to the alternative medicine sector is typically obtained from *L. latifolia* plants, which accumulate high levels of linalool, borneol, camphor, and 1,8-cineole, but no linalool acetate. The EOs obtained from the hybrid *L. x intermedia* plants contain a mixture of monoterpenes present in both parents, and are mainly utilized in personal care and hygiene products, such as soaps, shampoos, mouthwashes, as well as industrial and household cleaners, among others. *Lavandula x intermedia* plants produce up to 10 fold more EO than either parent, and are hence widely grown for EO production (Lis-Balchin, 2002; Upson and Andrews, 2004; Wells et al., 2018).

Metabolic engineering to enhance monoterpene biosynthesis has been reported in two lavender species, *L. latifolia* (spike lavender) and *L. x intermedia* (lavandin). The earliest metabolic engineering study in a lavender species was reported in the early 2000s, when Muñoz-Bertomeu et al. (2006) overexpressed the *Arabidopsis* 1-DXP synthase (DXS) gene in spike lavender. Like the earlier results reported for the overexpression of *DXR* and *DXS* in peppermint (Mahmoud and Croteau, 2001), *DXS* overexpression increased EO (monoterpene) production compared to untransformed controls. In another study, researchers attempted to enhance EO quality (scent) in *L. latifolia* plants by increasing the biosynthesis of *S*-linalool, a sweet-scented monoterpene (Mendoza-Poudereux et al., 2014). In this study, the *Clarkia breweri S*-linalool synthase gene was transformed into *L. latifolia* plants *via Agrobacterium*-mediated transformation. Transgenic lines overexpressing the transgene accumulated substantially (up to 1,000 fold) more *S*-linalool compared to untransformed control plants. Interestingly double transgenic plants, which resulted from a cross between separate transgenic plants overexpressing either DXS or *S*-linalool synthase gene (*LIS*) did not yield the expected results. Both essential oil yield and linalool content in double DXS-LIS transgenic plants were lower than that of their parents. A separate study also aimed at enhancing the fragrance of *L. x intermedia* essential oil by reducing 1,8-Cineole biosynthesis (Figure 3) by suppressing the expression of the 1,8-Cineole synthase gene using RNAi (Tsuro et al., 2019). The transcriptional expression of 1,8-Cineole synthase gene was suppressed in the transgenic plants compared to nontransgenic controls. RNAi was effective in reducing production of 1, 8-cineole significantly, and altering the overall scent of the EO of affected plants.

Finally, in a recent study, the *L. x intermedia* BPPS gene (*LiBPPS*) was placed under the control of the CaMV 35S promoter and stably expressed in transgenic *L. latifolia* plants *via Agrobacterium*-transformation in sense and antisense orientations (Adal et al., 2021, Unpublished). As expected, most plants expressing *LiBPPS* in sense produced more borneol and camphor (Figure 3), while those expressing *LiBPPS* in antisense accumulated less borneol and camphor than wild-type plants. Notably, the expression of *LiBPPS* in sense severely impeded the growth and development of most transformed plants, many of which (the highest transgene expressers) did not survive past early regeneration stages.

## Conclusion and Perspectives

In conclusion, the results of metabolic engineering efforts in mint and lavender have yielded useful information that paves the way for future investigation. The first lesson learned is that increasing precursor (IPP/DMAPP) through improving the output of the DXP pathway can help boost isoprenoid metabolism substantially in GTs. In this context, overexpression of limiting enzymes of the DXP pathway (DXS and DXR) individually has led to increased monoterpenoid production by up 350% (in spike lavender). The second lesson concerns manipulating the expression of TPS genes. Overexpression of TPSs sometimes leads to increase production of the corresponding terpene product. However, most often overexpression does not yield the desired (improved terpene production) results, clearly demonstrating that factors other than TPS expression are involved in isoprenoid production. Most of such (hypothetical) factors have not been yet defined. However, the use of GT-specific promoters – rather than constitutive promoters such as the CaMV 35S promoter – may help resolve some of the issues associated with ectopic overexpression of genes. Further, improving other factors potentially limiting isoprenoid biosynthesis, secretion and storage may result in enhanced isoprenoid production. For example, increasing the rate of the transport of the isoprenoids (presumably mediated by lipid transporters) from secretory cells into the storage cavity of the trichome may enhance isoprenoid production. Also, increasing the size and density of glandular trichomes may increase plant capacity to produce and store isoprenoid compounds. These approaches can be examined only when specific genes/proteins that control glandular trichome development and isoprenoid transport are identified.

## Author Contributions

SSM initiated and wrote the manuscript. SM and AMA contributed to searching and summarizing the articles and reviewing the manuscript. All authors contributed to the article and approved the submitted version.

### Conflict of Interest

The authors declare that the research was conducted in the absence of any commercial or financial relationships that could be construed as a potential conflict of interest.
